# Differences in *Haemophilus parasuis* adherence to and invasion of AOC-45 porcine aorta endothelial cells

**DOI:** 10.1186/1746-6148-9-207

**Published:** 2013-10-12

**Authors:** Rafael Frandoloso, Mateus Pivato, Sonia Martínez-Martínez, Elías F Rodríguez-Ferri, Luiz Carlos Kreutz, César B Gutiérrez Martín

**Affiliations:** 1 Section of Microbiology and Immunology, Department of Animal Health, University of León, León, Spain; 2 Laboratory of Microbiology and Immunology, Faculty of Agronomy and Veterinary Medicine, University of Passo Fundo, Passo Fundo, Brazil

**Keywords:** *Haemophilus parasuis*, Glässer’s disease, AOC-45 cells, Adherence, Invasion

## Abstract

**Background:**

The pathogenesis of *Haemophilus parasuis* depends on the bacterium’s ability to interact with endothelial cells and invade adjacent tissues. In this study, we investigated the abilities of eight *H. parasuis* reference strains belonging to serovars 1, 2, 4, 5, 7, 9, 10 and 13 to adhere to and invade porcine aortic endothelial cells (AOC-45 cell line).

**Results:**

The strains belonging to serovars 1, 2 and 5 were able to attach at high rates between 60 and 240 min of incubation, and serovars 4, 7 and 13 had moderate attachment rates; however, the strains belonging to serovars 9 and 10 had low adherence at all time points. Strong adherence was observed by scanning electron microscopy for the strains of serovars 5 and 4, which had high and moderate numbers, respectively, of *H. parasuis* cells attached to AOC-45 cells after 240 min of incubation. The highest invasiveness was reached at 180 min by the serovar 4 strain, followed by the serovar 5 strain at 240 min. The invasion results differed substantially depending on the strain.

**Conclusion:**

The reference strains of *H. parasuis* serovars 1, 2, 4 and 5 exhibited high adhesion and invasion levels to AOC-45 porcine aorta endothelial cells, and these findings could aid to better explain the pathogenesis of the disease caused by these serovars.

## Background

Glässer’s disease is a swine disease caused by a pleomorphic nicotinamide adenine dinucleotide-dependent Gram-negative rod of the family *Pasteurellaceae* known as *Haemophilus parasuis*[1]. The primary signs of this disease are serofibrinous to fibrinopurulent polyarthritis, polyserositis and meningitis, and septicemia, pneumonia and myositis of the masseter muscles are also observed [2]. To date, 15 *H. parasuis* serovars have been defined using an immunodiffusion test with heat-stable antigens [3]; however, some strains isolated from pigs with Glässer’s disease are untypable [4,5]. Serovars 1, 5, 10, 12, 13 and 14 have been classified as highly virulent; serovars 2, 4 and 15 as moderately virulent; and serovars 3, 6, 7, 8, 9 and 11 as non- or scarcely virulent [2], but the correlation between serotype and virulence is not clear [6].

Stress may influence the epidemiology of this disease within herds, especially regarding the early colonization of pigs by virulent strains and the spread of the disease throughout a swine population, especially in herds with high sanitary standards [2]. *H. parasuis* usually colonizes the upper respiratory tract of pigs, and it can also be detected in the tonsillar area and at other respiratory sites, such as the tracheal mucosa [7]. At these sites, virulent strains are able to breach the mucosal barrier and pass into the bloodstream, leading to the severe systemic disease described above [8]. Some *in vivo* studies have shown that *H. parasuis* does not strongly attach to cilia or other cell structures; in addition, the investigations into the specific interactions between this bacterium and host epithelial cells have been focused primarily on interactions with porcine brain microvascular endothelial cells [9-11] or porcine epithelial kidney cells [12]. In addition, cytolethal distending toxin, outer membrane protein 2 and heptose I and II residues in the inner core oligosaccharide have been implicated in the adherence of this bacterium to porcine alveolar macrophages, epithelial kidney cells and umbilicus vein endothelial cells [13-16].

To better understand the pathogenesis of Glässer’s disease, this study investigated the adherence to and invasion of AOC-45 porcine aortic endothelial cells by *H. parasuis* strains belonging to serovars 1, 2, 4 5, 7, 9, 10 and 13, which are considered to have different levels of virulence [2].

## Methods

### Bacterial strains

*H. parasuis* H409, H410, H412, Nagasaki, H643, H553, H555 and H793 (reference strains for serovars 1, 2, 4, 5, 7, 9, 10 and 13, respectively) were used. The bacteria were cultured on chocolate agar (BioMérieux, France) or in PPLO broth (Biolife, Italy) supplemented with 2.5 mg/ml glucose, 40 μg/ml nicotinamide adenine dinucleotide (Sigma) and 72.5 μg/ml IsoVitalex (Difco, USA).

### Determination of the infection dose

Because of the differences in the individual bacterial size, self-aggregation and growth time of the different *H. parasuis* strains, a growth curve for each strain was constructed to determine the infection dose. After the growth curves had been obtained (optical density -OD- versus colony-forming units -CFU-), the infection dose was established in the following manner: a mix of colonies from a fresh *H. parasuis* culture harvested from a chocolate plate were used to inoculate PPLO broth supplemented as described above, and this culture allowed to grow to an OD_600_ of 0.7. At this point, the bacteria were harvested by centrifugation at 4,000 × *g* for 15 min, washed three times in Dulbecco’s PBS medium (D-PBS) and resuspended in Dulbecco’s modified Eagle medium (DMEM) without antibiotics, and the concentration was adjusted to 10^8^ CFU/ml. Next, we prepared serial dilutions (1:10), and 100 μl of each dilution was plated on chocolate agar to determine the exact number of bacteria in the inoculum used for AOC-45 cell infection.

### Cell culture

AOC-45 aortic endothelial cells [17] were cultivated in DMEM (Invitrogen) supplemented with 10% fetal calf serum (Invitrogen) in 25 cm^2^ flasks (TPP, Switzerland) and incubated at 37°C in 5% CO_2_ and a 95% humid atmosphere. Cells were subcultured every 48 hours until 70% cell confluence was reached. Forty-eight-hour cultures (logarithmic phase of cell growth) were trypsinized and diluted in culture medium to obtain a concentration of 2 × 10^5^ cells per well in 24-well tissue culture plates (TPP, Switzerland).

### Measurement of the number of cell-associated bacteria

The adherence assay was performed as previously described [9], with some modifications. AOC-45 cells were grown to confluence in 24-well plates (approximately 7 × 10^5^ cells), washed once with 2 ml of D-PBS and infected with 1 ml aliquots of the different *H. parasuis* strains (10^8^ CFU). The plates were incubated for different times up to 240 min to allow bacterial adherence. Thereafter, the cells were washed five times with PBS to remove non-attached bacteria. To estimate the number of cell-associated bacteria (adhered to the cells surface and intracellular), the cells were incubated with 200 μl of trypsin for 5 min, followed by disruption with 800 μl of ice-cold deionized water and repeated pipetting to liberate cell-associated bacteria. Serial dilutions (1:10) of this cell lysate were prepared, and 100 μl of each dilution was plated onto chocolate agar (BioMérieux, France) and incubated for 48 h at 37°C with 5% CO_2_.

The average number of bacteria associated with each AOC-45 cell was determined by dividing the number of cell-associated bacteria harvested from chocolate plate by the number of AOC-45 cells in the well. These assays were performed in triplicate using directly thawed bacteria each time. The absolute number of AOC-45 cells in each well of each plate was determined by trypsinization and counting of an uninfected monolayer in a Neubauer chamber.

### Cell invasion assays

The invasion assays were performed as previously reported [9], with some modifications. Confluent monolayers of AOC-45 cells grown in 24-well plates were infected with 1 ml aliquots of the different *H. parasuis* strains (approximately 10^8^ CFU). The plates were centrifuged at 450 × *g* for 10 min to enhance the contact between *H. parasuis* and the surface of the monolayer. The plates were incubated for different times up to 300 min at 37°C with 5% CO_2_ to allow cell invasion by the bacteria. The monolayers were then washed as described for the adherence assays, and 1.5 ml of culture medium containing two antibiotics (5 μg of penicillin G/ml and 100 μg of gentamicin/ml, Sigma) was added to each well. Then, the plates were further incubated for 2 h at 37°C to kill the extracellular *H. parasuis*. The monolayers were washed thrice, and the cells were disrupted as described for the adherence assays. Serial dilutions of this cell lysate (100 μl) were plated onto chocolate agar and incubated for 48 h at 37°C with 5% CO_2_.

The level of invasion was determined by dividing the number of bacteria harvested from the chocolate plate by the number of AOC-45 cells in the well. The assays were performed in triplicate as indicated above. The absolute number of AOC-45 cells in each well of each plate was determined as described for the adherence assay.

### Adherence and invasion analyses using electron microscopy

The adherence *H. parasuis* serovars 4, 5 and 9 was also assessed using scanning electron microscopy (SEM). AOC-45 cells were grown on 13 mm Thermanox coverslips in a 24-well culture plate until confluence was reached (approximately 7 × 10^5^ cells). Then, the cells were infected as described above and incubated for 240 min at 37°C. After five washes with 2 ml of D-PBS, the monolayers were fixed for 1 h at 4°C with 2% glutaraldehyde in 0.1 M cacodylate buffer (pH 7.3). After three other washes with cacodylate buffer at 4°C for 10 min each, the samples were postfixed for 15 min at room temperature with 2% osmium tetroxide in deionized water. The specimens were dehydrated in a graded series of ethanol solutions and desiccated in a critical point dryer apparatus (Bal-Tec CPD 030) [18]. After an ion-spatter coating with gold-palladium, the samples were viewed with a JSM-6480 JEOL scanning electron microscope.

For invasion studies, transmission electron microscopy (TEM) was used. Cells grown on Thermanox coverslips were infected as described above for the adherence assays and processed as previously described [12].

### Statistical analysis

The results are presented as the mean ± standard deviation. Analysis of variance (ANOVA) and Tukey’s multiple comparison tests (SPSS statistical program, version 16.0, Chicago, IL, USA) were used to compare adherence or invasion after different incubation times and to compare adherence or invasion between the different strains. GraphPad Prism, version 5.0 (San Diego, CA, USA), was used to prepare the figures. *P* values <0.05 indicate statistical significance.

## Results

### Cell-associated bacteria

**Figure 1:**
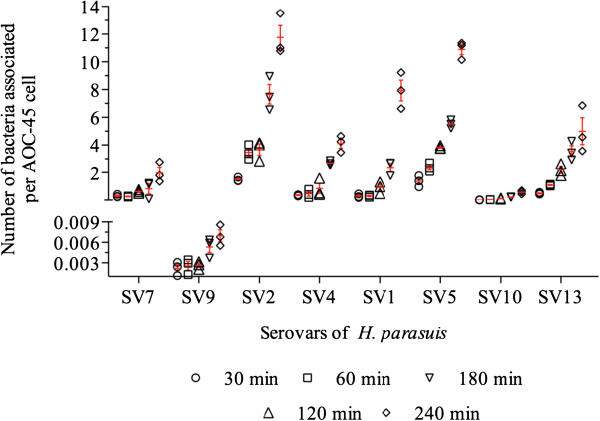
**Adherence of different *H. parasuis *strains of different serovars to AOC-45 cells from 30 to 240 minutes of incubation.** Each value is the mean ± standard deviation of three assays.

All the analyzed *H. parasuis* strains were able to adhere to AOC-45 cells in a time-dependent manner (Figure 1). The highest numbers of cell-associated bacteria after 30 min of incubation were observed for the strains of serovars 2 (1.51 ± 0.13) and 5 (1.38 ± 0.41). The lowest level of adhesion at this time was observed for the strains of serovar 9 (0.002 ± 0.0009). At the longest time compared (240 min), the greatest level of adherence was observed for the strains of serovars 2 (11.04 ± 2.3), 5 (10.90 ± 0.6) and 1 (7.6 ± 1.8), followed by those of serovars 13 (4.67 ± 1.8) and 4 (4.12 ± 0.6). The attachment rate of the serovar 7 strain at this time was significantly lower (1.98 ± 0.7, *P* < 0.05) than that observed for the strains of serovars 2, 4, 5 and 13. The attachment rates of serovars 10 (0.58 ± 0.1) and 9 (0.006 ± 0.001) were approximately 1,800 times lower than those observed for the strains of serotypes 2 and 5 (*P* < 0.05).

The adherence of the most strains increased considerably at 180 min and reached the highest level at 240 min. Significant differences were observed for the strains of serovars 1, 2, 5 and 13 between the levels of adherence at 180 min and 30 min (*P* < 0.05). Of the four *H. parasuis* serovars with high virulence (1, 5, 10 and 13) according to Oliveira and Pijoan [2], the serovar 5 strain exhibited the highest rate of adherence at 30 min, with significant differences between this strain and the strains of serovars 1, 10 and 13 (*P* < 0.05). No significant difference was found between the strains of serovars 1 and 13 at this time, but both presented higher adherence levels than those of serovar 10 (*P* < 0.05). After 180 min of incubation, the strains of serovars 1 and 5 had significantly more adherent bacteria (3.2 and 2-fold greater, respectively) than at earlier times (Figure 1). Of the two moderately virulent serovars [2], the serovar 2 strain clearly exhibited a significantly higher adherence capacity than the serovar 4 strain (*P* < 0.05) throughout the study. The adherence kinetics of the strains of serovars 2 and 5 were similar, and a significant difference (*P* < 0.05) could be seen between these strains only at the 180 min time point (Figure 1).

**Figure 2:**
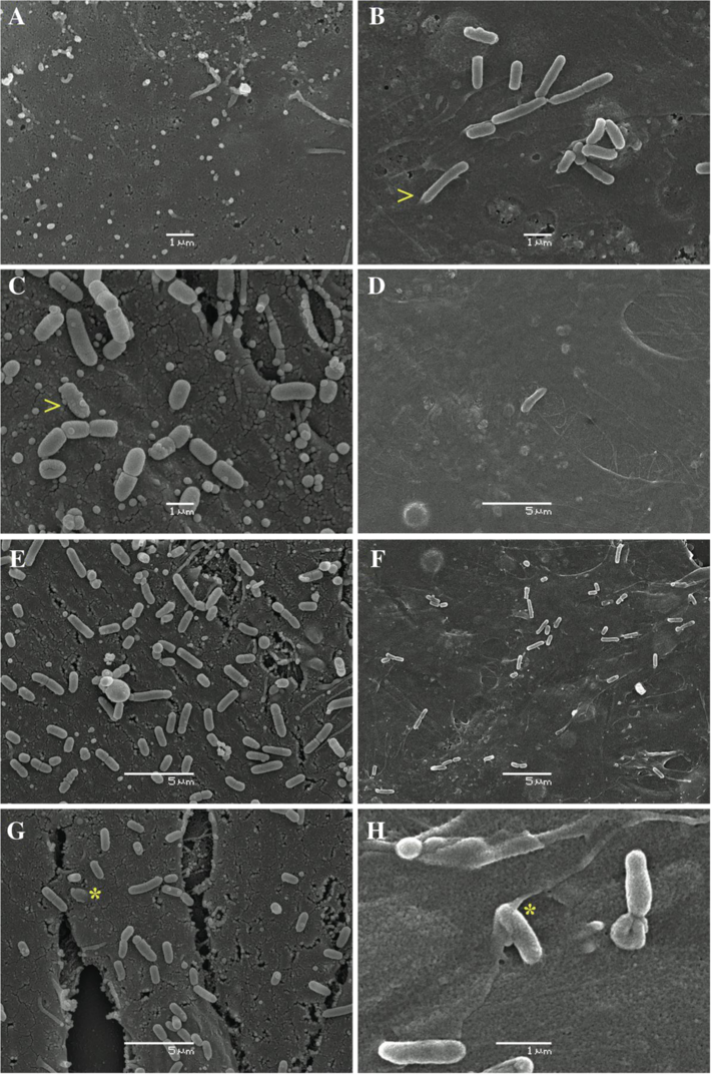
SEM micrographs showing (A) uninfected AOC-45 cells, (B) *H. parasuis *serovar 4 strain adhered to AOC-45 cells, (C) *H. parasuis *serovar 5 strain adhered to AOC-45 cells, (D) a small number of serovar 9 strain *H. parasuis *cells adhered to AOC-45 cells, (E) a high number of serovar 5 strain *H. parasuis *cells adhered to AOC-45 cells, (F) a moderate number of serovar 4 strain *H. parasuis *cells adhered to AOC-45 cells, (G) a bacterium belonging to the *H. parasuis *serovar 5 strain projected into AOC-45 cells, as indicated by the asterisk (*) and (H) a bacterium belonging to the *H. parasuis *serovar 4 strain invading AOC-45 cells, as it is indicated by the asterisk (*). Concentration of the bacterial inoculum: approximately 10^8^ CFU. Incubation time: 240 min. Morphological differences between the strains of serovars 4 and 5 are indicated by (>).

SEM was used to confirm the adhesion of the strains of serovars 4, 5 and 9 after 240 min of incubation. In absence of bacteria, AOC-45 cells had an irregular surface (Figure 2A), and no morphological changes were observed in these cells after incubation with these serovars. The adherence of strains of serovars 4, 5 and 9 is shown in Figures 2B, 2C and 2D, respectively. The number of bacteria attached to the cell surface was higher for the serovar 5 strain (Figure 2E) than for the serovar 4 strain (Figure 2F), in agreement with the results obtained with the adherence assays in 24-well plates (Figure 1). Morphological differences between the strains of serovars 4 and 5 were observed: the serovar 4 strain (Figure 2B) was a longer and narrower rod (approximately 2 μm) than the serovar 5 strain (approximately 1 μm, Figure 2C). The invasion of AOC-45 cells by the strains of serovars 5 and 4 is shown in Figures 2G and 2H, respectively.

### Invasion

**Figure 3:**
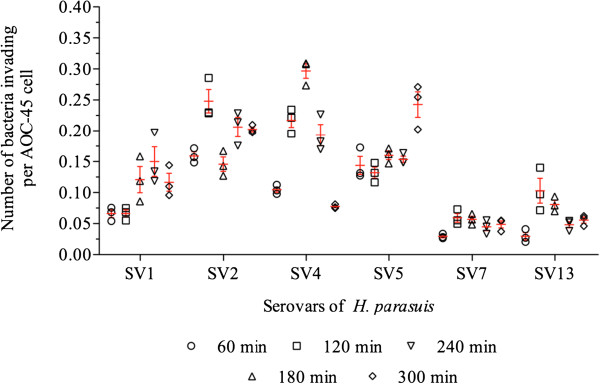
**Invasion of AOC-45 cells by different *H. parasuis *strains of different serovars between 60 and 300 minutes of incubation.** Each value is the mean ± standard deviation of three assays.

The invasion assays revealed differences among the strains (Figure 3). Of the serovar 7 and 9 strains, which were classified as non-virulent by Oliveira and Pijoan [2], only the serovar 7 strain was able to invade AOC-45 cells, though only at low numbers. The highest level of invasion was detected at 120 min (0.05 ± 0.001), and the level of invasion at this time was significantly greater (*P* < 0.05) than that observed at 60 min (Figure 3). As observed for the serovar 9 strain (data for this strain are not shown), the strain of serovar 10 was not able to invade aortic cells.

The serovar 5 strain was the only strain for which invasion was time dependent, with the lowest level of invasion being detected at 120 min (0.13 ± 0.01) and the highest at 300 min (0.24 ± 0.03). The five other strains exhibited invasion kinetics patterns that were completely different. For instance, the highest invasion rate for the strain of serovar 1 was recorded at 240 min (0.15 ± 0.04), whereas that highest invasion rate for serovar 4 was at 180 min (0.29 ± 0.02) and those of serovars 2 (0.24 ± 0.03), 7 (0.05 ± 0.01) and 13 (0.1 ± 0.03) were at 120 min.

The analysis of the highly virulent serovars [2] showed that the strain of serovar 5 had significant higher invasion rates at 60 and 300 minutes (*P* < 0.05) than the strains of serovars 1 and 13. No significant differences were found between the invasion rates of the strains of serovars 1 and 13 from 60 to 180 min; however, the serovar 1 strain had significantly greater invasiveness than the serovar 13 strain at 240 and 300 min (*P* < 0.05).

With respect to the invasion kinetics of the moderately virulent serovars [2], the strain of serovar 4 had a significantly higher invasion rate at 180 min than the serovar 2 strain (*P* < 0.05). However, after this time point, a sharp decrease was observed for the invasiveness of serovar 4, with the level of invasion at 5 h being approximately three-fold lower than that of serovar 2 (*P* < 0.05) (Figure 3).

**Figure 4:**
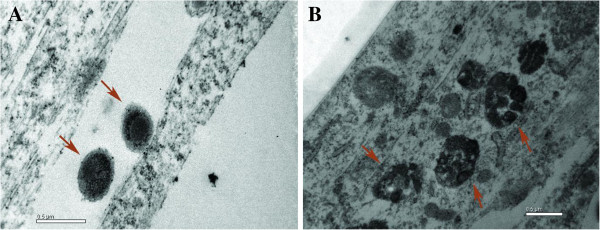
**The serovar 4** ***H. parasuis *strain adheres to (A) and invades (B) AOC-45 cells (TEM micrographs).** Concentration of the bacterial inoculum: 10^8^ CFU. Incubation time: 300 min.

TEM was used to support the invasion results. The strains of serovars 4 and 5 were observed by microscopy after 180 and 300 min of incubation, respectively (data for the strain of serovar 5 are not shown). At these times, we observed the intracellular invasion of AOC-45 cells by *H. parasuis*, as observed in the cell invasion assays in 24-well plates. Figure 4A shows a bacterium of serovar 4 in close contact with the cellular membrane of an AOC-45 cell, and Figure 4B shows a high number of bacteria of the same serovar in the cytoplasm of these cells.

## Discussion

*H. parasuis* adherence and invasion were evaluated for eight reference strains belonging to serovars with different virulence levels [2]. A well-established endothelial cell line of vascular origin (AOC-45) was used, and although these cells differ from the epithelial cells lining the respiratory tract, this endothelial cell line could help explain some of the virulence mechanisms implied in the pathogenicity of this porcine pathogen. Although extrapolation of findings in other cell lines must be taken with caution, cytotoxicity studies were not carried out because in a previous report [10] using *H. parasuis* and another vascular endothelial cell line, no apoptotic effect was detected for up to 5 hours post infection, the last time tested in our studies.

Our results clearly indicate that all eight strains were able to adhere to porcine aortic cells *in vitro*, although to considerably different degrees depending on the strain and serovar. However, the strains of serovars 9 and 10, which had the lowest adherence abilities, were not able to invade AOC-45 cells. Because of the use of a centrifugation step in the invasion assays but not in the adherence assays in this study, the number of *H. parasuis* able to invade these cells could be overestimated compared with the number of adhered bacteria. This centrifugation step was included to facilitate contact between the bacteria and the AOC-45 cells in a short time, eliminating the normal effect of gravity. Even with the use of this step, the number of *H. parasuis* bacteria associated with AOC-45 cell surfaces observed in this study was similar to that found previously [9] for the porcine brain microvascular endothelial line PBMEC/C1-2.

The attachment rate obtained in our study for the strain belonging to serovar 5 was higher than those previously reported [12]. More than twice as many *H. parasuis* cells (5.52 ± 0.2) adhered to AOC-45 cells after 180 min of incubation than adhered to PK-15 cells (approximately 2.1 ± 0.2). This result could indicate *H. parasuis* tropism for endothelial cells, such as AOC-45 cells, because this organism has to adhere to endothelial cells, then cross through blood vessels and finally reach the target organs for infection. This finding could explain the bacterium’s ability to cause a severe systemic inflammatory response, as inflammatory lesions in porcine hearts have been reported [13]. In studies based on a clinical isolate belonging to serovar 4, the adherence to and invasion of PK-15 cells and PUVECs were found to be related, respectively, to lipooligosaccharide heptose residues [14], cytolethal distending toxin [15] and Omp2 [16].

The adhesion of serovar 5 to kidney epithelial [12] and brain microvascular endothelial cells [9] reached the highest percentages after 90 min of incubation; however, the attachment exhibited by the eight strains used in this study was time dependent, and the greatest values were measured after 240 min of incubation, the longest time tested, irrespective of the adherence degree of each strain.

Microscopically, no morphological changes to the aortic cells were detected by SEM after the attachment of the different *H. parasuis s*erovars. This result is in agreement with those for brain endothelial cells after 30 min of infection [9]. Considerable changes in the surface of porcine kidney epithelial cells have been detected during *H. parasuis* attachment, including an increase in the number of membranous projections [12]. The formation of these structures was hypothesized to be one of the mechanisms involved in the adherence of this strain to PK-15 cells. Further studies are required to determine which molecules are involved in the adherence of *H. parasuis* to available porcine cell lines. The adherence exhibited by the strains belonging to serovars 1, 2 and 5 in this study could indicate that a high adherence strength is necessary to prevent *H. parasuis* from being dragged along by the arterial or venous bloodstream, thus facilitating the infection of target tissues. Similarly, the most clinically important serovars were those with the greatest *in vitro* adherence levels starting from 30 min of infection in our study. This fact, associated with the capacity of *H. parasuis* to adhere to porcine epithelial tracheal cells [19], could suggest that vaccine design strategies should focus on eliciting not only a systemic immune response but also a strong mucosal immune response, so that *H. parasuis* would not be able to adhere to epithelial cells and then reach the bloodstream.

Although *H. parasuis* was previously described as an exclusively extracellular pathogen, other investigations [9] have proven that this organism is able to cross the blood–brain barrier and invade brain endothelial cells. Similarly, we showed that several *H. parasuis* strains belonging to different serovars were able to invade other type of endothelial cells *in vitro*, a process also recently demonstrated for PUVECs [14-17]. Our findings revealed the high attachment and invasiveness of virulent strains belonging to serovars 1 and 5 (H409 and Nagasaki, respectively) when using the AOC-45 cell line; however, strains of other serovars also considered to be virulent [2] attached in a lesser degree, such the strain of serovar 13, and the strain of serovar 10 was incapable of invading these cells. The invasiveness exhibited by the reference strains of serovars 1, 2, 4 and 5 used in this study could aid to explain the ability of these serovars to cause clinical cases of systemic disease [20].

In a previous study [19], 10^7^ CFU of Nagasaki strain (serovar 5) was able to reach the intracellular space of the brain endothelial cells after 90 min of incubation. Our results indicate that 10^8^ CFU of *H. parasuis* strains of serovars 2 or 4 was able to invade a higher proportion of AOC-45 cells at this same time. One hypothesis to explain this finding is that the strains with high invasiveness could use this mechanism to pass through endothelial cells and reach the target organs. Some authors [9,21] have shown that the number of internalized *Pasteurella multocida* and *H. parasuis* decreased in a time-dependent manner, and they suggested that an exocytosis-based mechanism could explain this behavior. This fast mechanism could explain also, generically, the low number of internalized bacteria recovered in our study.

More studies should be performed to test this hypothesis and to determine whether the cytoplasmatic conditions and innate defense mechanisms of AOC-45 cells are compatible with *H. parasuis*. The invasion of AOC-45 cells by *H. parasuis* followed a heterogeneous pattern in our study and depended on the strain and serovar, unlike the results of other authors [11], who did not find differences in the ability to invade associated with the *H. parasuis* serovar.

## Conclusion

The reference strains of *H. parasuis* serovars 1, 2, 4 and 5, which are thought to be highly or moderately virulent, had high adhesion and invasion levels to AOC-45 porcine aorta endothelial cells, while the reference strain of serovar 9, thought to have low virulence potential, attached poorly to these cells. These findings could aid to better explain the pathogenesis of Glässer’s disease caused by serovars 1, 2, 4 and 5. Further studies with clinical isolates are required to confirm our findings.

## Abbreviations

AOC: Aorta endothelial cells; OD: Optical density; CFU: Colony-forming units; D-PBS: Dulbecco’s PBS; SEM: Scanning electron microscopy; TEM: Transmission electron microscopy; ANOVA: Analysis of variance; PUVECs: Porcine umbilicus vein endothelial cells.

## Competing interests

The authors declare that they have no competing interests.

## Authors’ contributions

RF contributed to the conception and design of the study, as well as the data analysis and drafting of the manuscript; MP and SMM contributed to the adherence and invasion studies; LCK contributed to the critical revision of the manuscript; CBGM contributed to the data analysis and writing of the manuscript; and EFRF contributed to the critical revision and final approval of the manuscript. All authors have read and approved the final manuscript.
